# Artificial intelligence (AI)—it’s the end of the tox as we know it (and I feel fine)*

**DOI:** 10.1007/s00204-023-03666-2

**Published:** 2024-01-20

**Authors:** Nicole Kleinstreuer, Thomas Hartung

**Affiliations:** 1 NIH/NIEHS/DTT/NICEATM, Durham, NC USA; 2 Bloomberg School of Public Health, Doerenkamp-Zbinden Chair for Evidence-Based Toxicology, Center for Alternatives to Animal Testing (CAAT), Johns Hopkins University, Baltimore, MD USA; 3 CAAT-Europe, University of Konstanz, Constance, Germany

**Keywords:** Artificial intelligence, Toxicology, Predictive modeling, Chemical safety, Risk assessment, Computational toxicology

## Abstract

The rapid progress of AI impacts diverse scientific disciplines, including toxicology, and has the potential to transform chemical safety evaluation. Toxicology has evolved from an empirical science focused on observing apical outcomes of chemical exposure, to a data-rich field ripe for AI integration. The volume, variety and velocity of toxicological data from legacy studies, literature, high-throughput assays, sensor technologies and omics approaches create opportunities but also complexities that AI can help address. In particular, machine learning is well suited to handle and integrate large, heterogeneous datasets that are both structured and unstructured—a key challenge in modern toxicology. AI methods like deep neural networks, large language models, and natural language processing have successfully predicted toxicity endpoints, analyzed high-throughput data, extracted facts from literature, and generated synthetic data. Beyond automating data capture, analysis, and prediction, AI techniques show promise for accelerating quantitative risk assessment by providing probabilistic outputs to capture uncertainties. AI also enables explanation methods to unravel mechanisms and increase trust in modeled predictions. However, issues like model interpretability, data biases, and transparency currently limit regulatory endorsement of AI. Multidisciplinary collaboration is needed to ensure development of interpretable, robust, and human-centered AI systems. Rather than just automating human tasks at scale, transformative AI can catalyze innovation in how evidence is gathered, data are generated, hypotheses are formed and tested, and tasks are performed to usher new paradigms in chemical safety assessment. Used judiciously, AI has immense potential to advance toxicology into a more predictive, mechanism-based, and evidence-integrated scientific discipline to better safeguard human and environmental wellbeing across diverse populations.

## Introduction

Artificial intelligence (AI) refers to computer systems that are capable of performing tasks that typically require human intelligence, such as visual perception, speech recognition, decision-making, and language translation (Pérez-Santín et al. 2021). AI has seen rapid advancements in recent years, driven by exponential growth in computing power, availability of large datasets, and improvements in machine learning algorithms. The field of toxicology is poised to benefit immensely from the integration of AI techniques (e.g., Luechtefeld and Hartung 2017; Idakwo et al. 2018; Tang et al. 2018; Baskin 2018; Luechtefeld et al. 2018a, Bhhatarai et al. 2019; Pu et al. 2019; Mansouri et al. 2021; Lin and Chou 2022; Jeong et al. 2022; Sedykh et al. 2022; Tuyet et al. 2023, Hartung 2023b, 2023c).

**Fig. 1:**
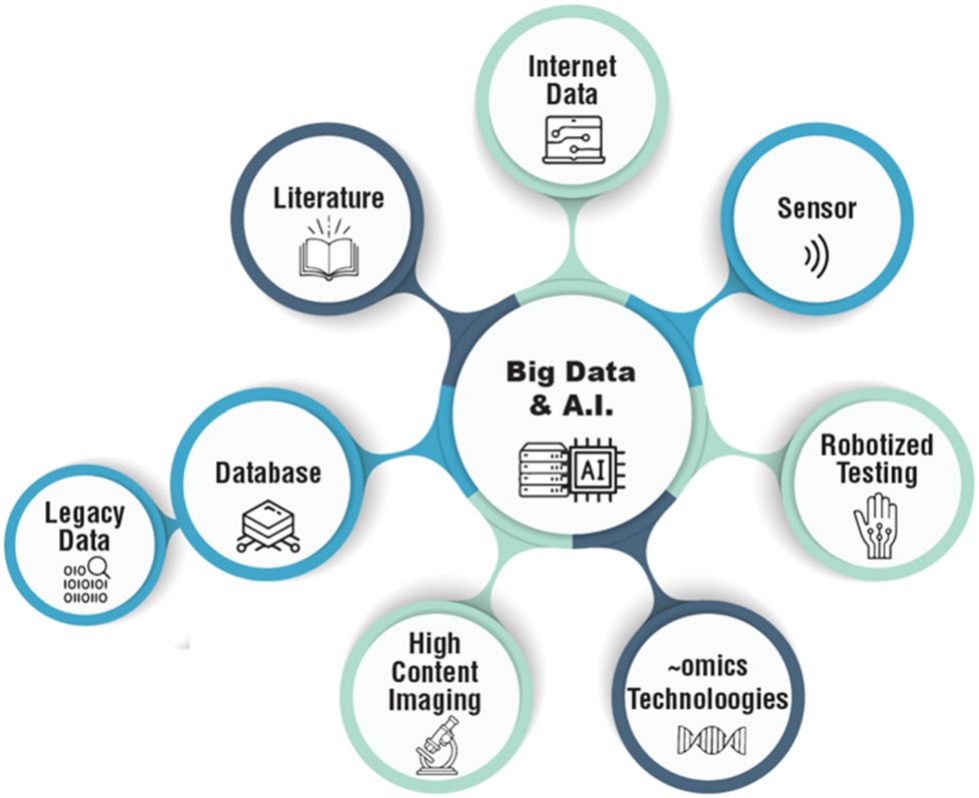
A variety of data sources transform toxicology from a data-poor to a data-rich discipline

Toxicology deals with understanding the harmful effects of chemical, physical and biological agents on living organisms and the ecosystem. It involves generating, integrating, and analyzing data from diverse sources to predict toxicity potentials, determine mechanisms of action, and enable risk assessment of toxins and toxicants (Wu and Wang 2018; Lin and Chou 2022). While the majority of AI use so far is in assessing human health risks, ecotoxicology is following suite (Miller et al. 2018; Wu et al. 2022). AI is well suited to handle the complexity and large volumes of data associated with modern toxicology research (Fig. 1). In particular, machine learning, a subset of AI focused on algorithms that can learn from data and generate novel predictions, has emerged as a promising approach with diverse applications in toxicology (Wu and Wang 2018). The last decade has seen dramatic increases in the generation, publication, and accessibility of scientific information, resulting in an AI-enabling resource known as “big data” (Shilo et al. 2020).

**Fig. 2:**
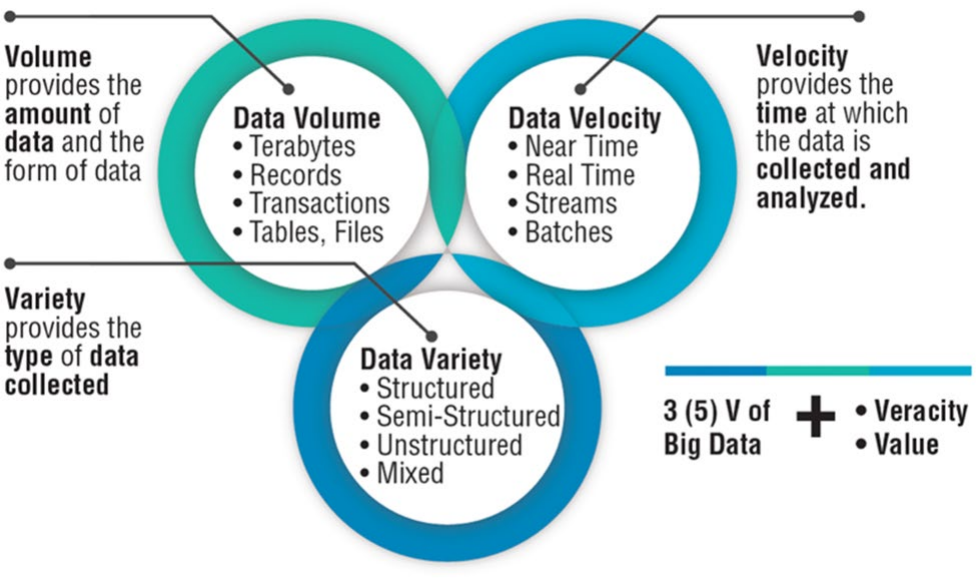
The Three (or Five) V defining Big Data. Modified and redrawn from https://www.rd-alliance.org/group/big-data-ig-data-development-ig

Big data is a term that describes large volumes of data—both structured and unstructured—that form the basis of AI technologies. A simple definition of AI could be: technologies to make big sense from big data. However, the amount of data alone is not the sole driver, but this concept is more comprehensively defined by the Five-V (Fig. 2):

1. Volume: The quantity of data that is produced is a critical aspect of big data, and has consequences in terms of storage, management, and energy consumption. The volume could range from dozens of terabytes to several petabytes in a single data set.
2. Velocity: This refers to the speed at which data is created, stored, analyzed, and visualized. In many situations, data need to be analyzed in real-time or near real-time to provide value.
3. Variety: This term refers to the many types of data that are available. Traditional data types were structured and could easily fit into a relational database. With the rise of big data, data types are now classified as structured, semi-structured, and unstructured.
4. Veracity: This term refers to the quality of the data. Because data come from various sources, it is important to test the veracity/quality of the data.
5. Value: This refers to our ability to analyze, interpret, translate, and apply data to gain insights that can help advance knowledge and improve decision making, and thereby increase value.

**Fig. 3:**
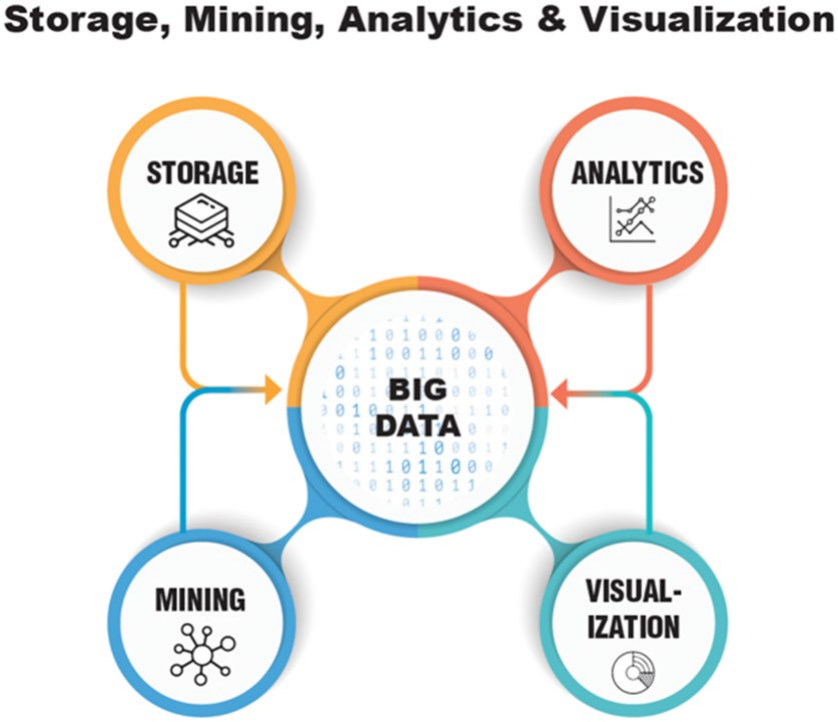
The principal tasks of handling Big Data. Modified and redrawn from https://www.edureka.co/blog/top-big-data-technologies

Big data technology allows for the storage, processing, mining, and analysis of these large datasets (Fig. 3). Apache Hadoop and Spark are popular frameworks used for big data processing, and they allow distributed processing of large datasets across clusters of computers. Big data analytics involves the use of analytics techniques like machine learning, data mining, natural language processing, and statistics.

**Fig. 4:**
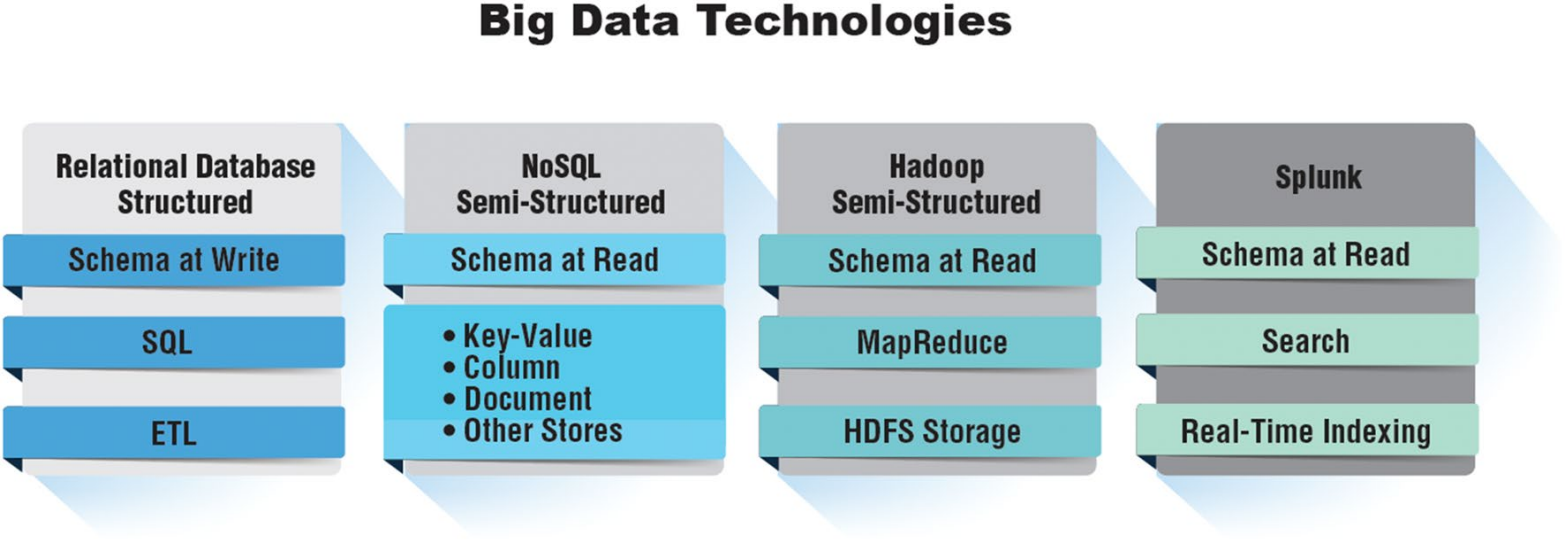
Example Big Data technologies moving from highly structured to increasingly unstructured approaches. Modified and redrawn from https://www.slideshare.net/Splunk/splunksummit-2015-real-world-big-data-architecture. SQL (Structured Query Language) is a programming language used to communicate with data stored in a relational database management system. ETL, which stands for extract, transform and load, is a data integration process that combines data from multiple data sources into a single, consistent data store that is loaded into a data warehouse or other target system. NoSQL databases (aka "not only SQL") are non-tabular databases and store data differently than relational tables. NoSQL databases come in a variety of types based on their data model. The main types are document, key-value, wide-column, and graph. They provide flexible schemas and scale easily with large amounts of data and high user loads. The Apache Hadoop software library is a framework that allows for the distributed processing of large data sets across clusters of computers using simple programming models. MapReduce is a programming model or pattern within the Hadoop framework that is used to access big data stored in the Hadoop File System (HDFS), i.e., the primary data storage system used by Hadoop applications. Splunk is a big data platform that simplifies the task of collecting and managing massive volumes of machine-generated data and searching for information within it

Until recently, databases of the past had to be well-structured as Relational Databases to allow analyses and struggled with incomplete datasets. However, increasingly unstructured datasets are employed, and structuring is part of the analytical algorithm itself (Fig. 4).

**Fig. 5:**
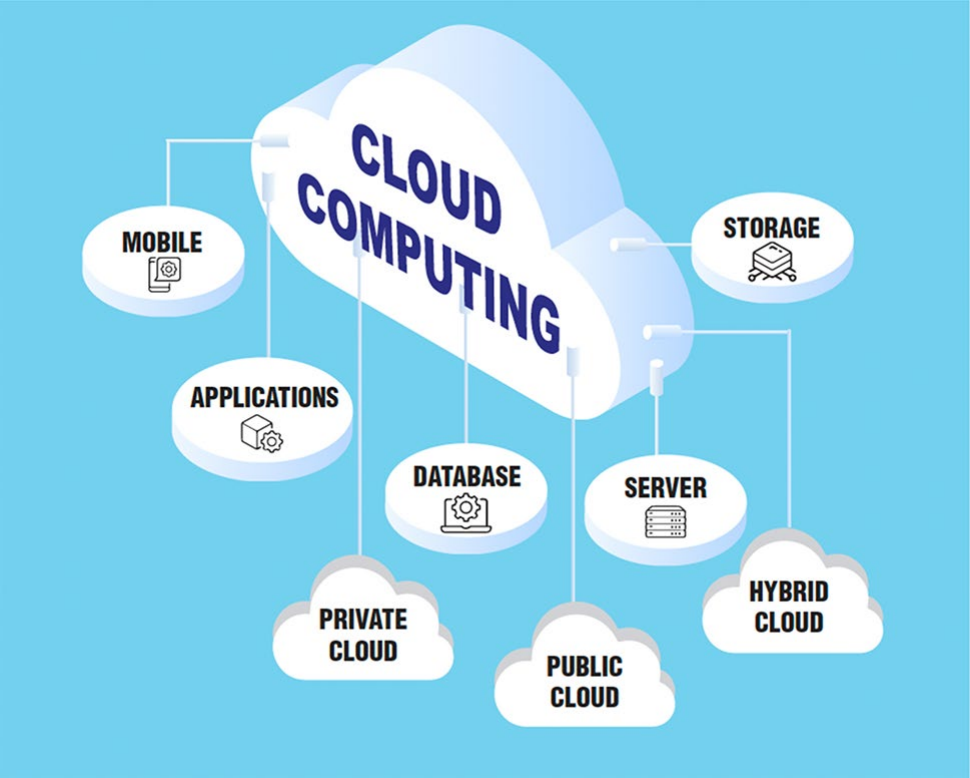
Cloud computing often makes big data approaches easier but is not a prerequisite. Modified and redrawn from https://medium.com/mycloudseries/how-to-start-using-cloud-computing-as-a-startup-77055c60f74f

Cloud computing (Fig. 5) has made many Big Data approaches easier, but it is not at all a prerequisite for AI.

## AI Impact on toxicology

Some key areas where AI is expected to transform toxicology include:

1. **Predictive toxicology** Machine learning models can be trained on existing datasets of chemicals and their toxicity profiles to predict potential toxicity of new chemical entities. This can accelerate chemical screening and reduce reliance on animal testing (Luechtefeld et al. 2018a; Mansouri et al. 2020, 2021).
2. **Data analysis** AI techniques like natural language processing can automate tasks like mining legacy animal studies, extracting information from scientific literature, analyzing high-throughput screening data, and integrating diverse omics datasets (Kavlock et al. 2012; Foster et al. 2024).
3. **Risk assessment** AI models provide probabilistic outputs that account for uncertainty and variability, enabling more robust quantitative risk assessment (Zhang et al. 2019; Gilmour et al. 2022).
4. **Mechanistic research** Although AI models are often “black-boxes”, advances in explainable AI can help provide insight into mechanisms underlying chemical toxicity (Kleinstreuer et al. 2018, 2020).
  This review aims to provide a comprehensive overview of the emerging applications of AI in predictive toxicology, data analysis, risk assessment, and mechanistic research. It summarizes key accomplishments, challenges and opportunities at the intersection of AI and toxicology, and provides specific examples of projects and tools that are enabling innovative applications of AI. The review concludes with recommendations for integrating and advancing AI in a responsible manner to transform the field of toxicology.

## Introduction into AI in the life sciences

**Fig. 6:**
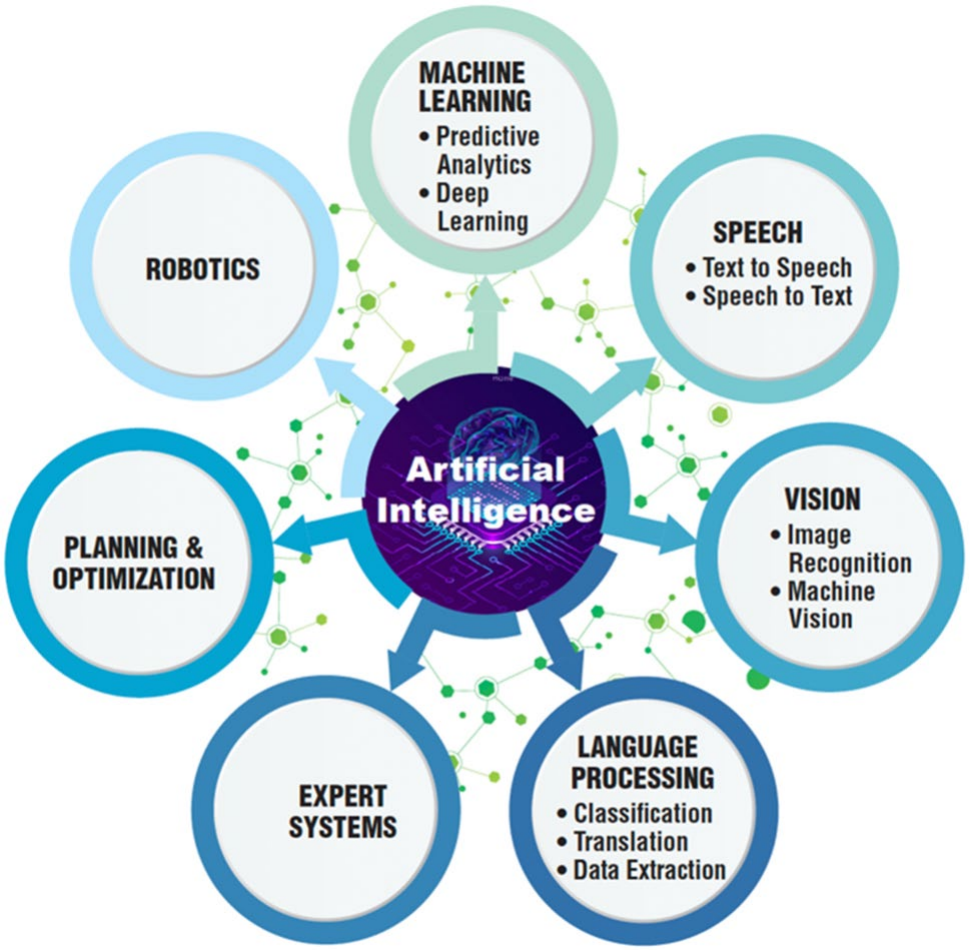
Different forms of Artificial Intelligence (AI) uses. Modified and redrawn from https://www.datamation.com/artificial-intelligence/what-is-artificial-intelligence/

Artificial Intelligence (AI) is a rapidly developing field with tremendous impact on various domains of the life sciences. AI comes in many different forms (Fig. 6).

**Fig. 7:**
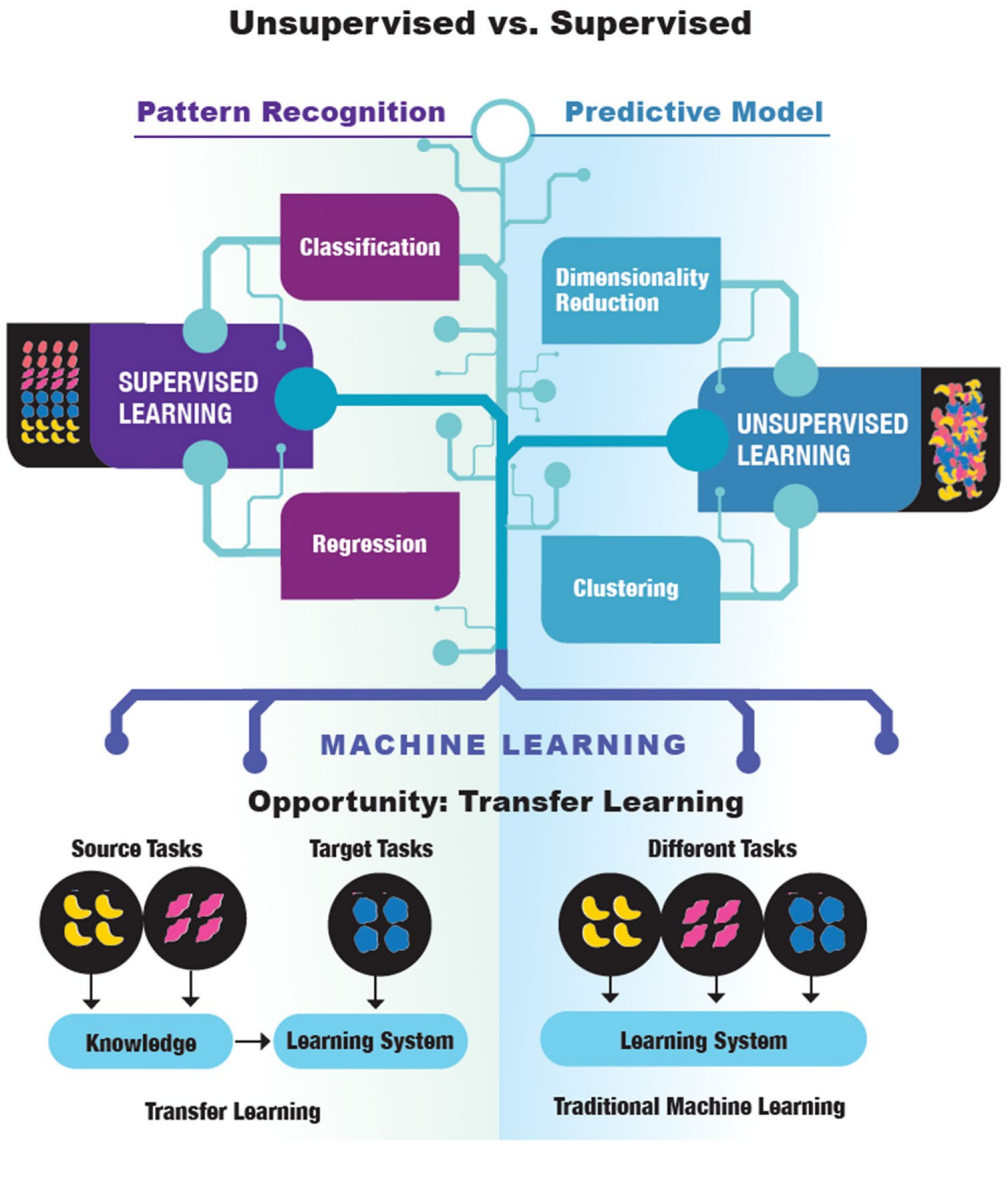
Both supervised and unsupervised learning can benefit from transfer learning. Modified and redrawn from https://i.ytimg.com/vi/mKTD8X4iokQ/maxresdefault.jpg and https://www.semanticscholar.org/paper/A-Survey-on-Transfer-Learning-Pan-Yang/a25fbcbbae1e8f79c4360d26aa11a3abf1a11972/figure/0

**Fig. 8:**
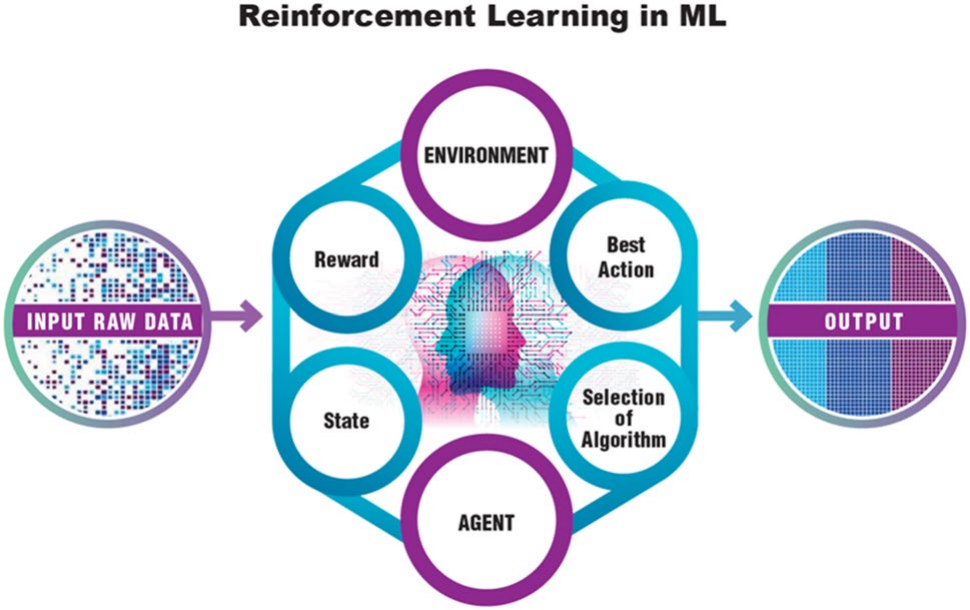
The principles of reinforcement learning. Reinforcement learning represents a branch of machine learning where an agent (acting entity) determines how to make decisions by performing actions within a specific environment to amplify a cumulative reward metric. Through a process of trial and error, the agent experiences penalties or rewards for its actions, aiming to formulate a policy, essentially a guide to deciding what action to take in a given scenario. Modified and redrawn from https://dynagrace.com/what-is-reinforcement-learning-all-you-need-to-know-about/

**Fig. 9:**
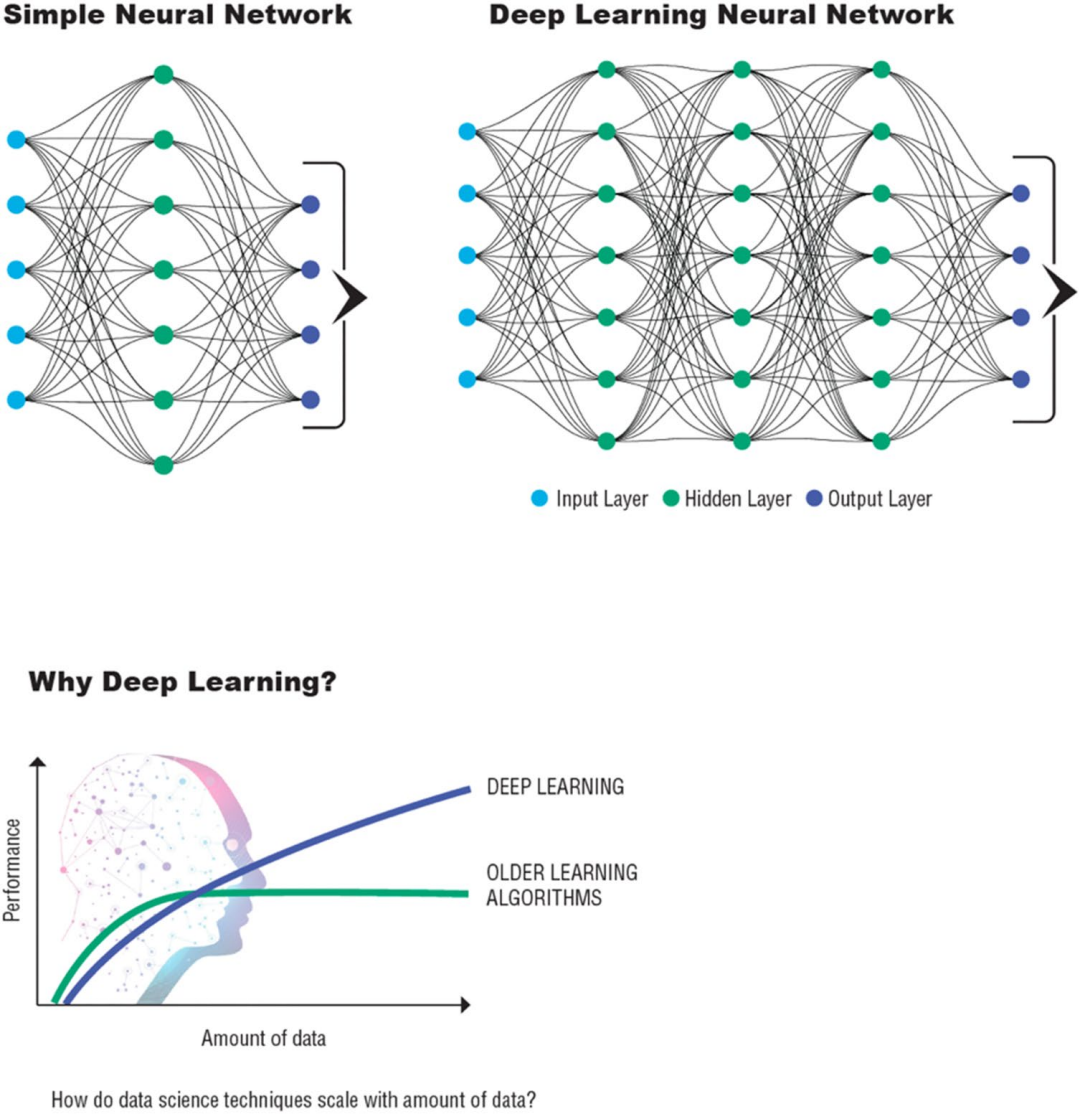
The principles of deep learning. Deep learning is a form of machine learning, which involves the use of artificial neural networks with several layers—hence the term "deep"—to model and understand complex patterns in datasets. DL continues to become better with increase in data. Modified and redrawn from https://verneglobal.com/news/blog/deep-learning-at-scale and https://towardsdatascience.com/is-deep-learning-hitting-the-wall-d2f560419daf

Life scientists can leverage the power of AI to analyze vast and complex biological data sets, enhance the precision and speed of diagnosis, expedite drug discovery, personalize medicine, elucidate disease mechanisms, and much more in a truly transformative way for healthcare,^1^,^2^ (Hartung 2023c). AI is the ability of a digital computer or machine to perform tasks commonly associated with intelligent beings. This includes learning from experiences, understanding natural language, recognizing patterns, and making decisions. Central concepts include:

1. Machine Learning (ML)—ML is a subset of AI that uses statistical methods to enable machines to improve with experience. This is particularly useful in life sciences, where vast amounts of data are generated. For instance, ML can help identify disease patterns from huge genomic datasets or medical images. Multiple types of learning can be distinguished.
  - In Supervised Learning (Fig. 7, right side), the model is trained on a labeled dataset. That is, each instance in the training set includes both the input data and the correct output, often called the label or the target. The goal of the model is to learn a function that, given an input, predicts the correct output. Common tasks include regression (predicting a continuous output) and classification (predicting discrete categories).
  - In Unsupervised Learning (Fig. 7, left side), the model is trained on a dataset without labels, and the goal is to discover structure in the data. This could involve clustering, where the aim is to group similar instances together, or dimensionality reduction, where the aim is to simplify the data without losing too much information. Another common task is anomaly detection, where the aim is to detect unusual instances in the data. Both supervised and unsupervised learning techniques can leverage transfer learning to improve algorithmic outcomes.
  - Transfer learning (Fig. 7, lower panel) is a ML technique where a pre-trained model, often developed for a large-scale task like image recognition or language understanding, is used as the starting point for a related but different task. The idea is to leverage the patterns and knowledge learned from the first task, which had abundant data, to improve performance on the second task that may have less available data. This approach can significantly reduce training time and computational resources, and often yields better performance, especially in scenarios where training data is limited.
  - Reinforcement Learning (Fig. 8) is a type of ML where an agent learns to make decisions by taking actions in an environment to maximize some notion of cumulative reward. The agent learns from trial and error, receiving rewards or penalties for the actions it performs, and its goal is to learn a policy, which is a strategy that dictates what action to take under what circumstances. It is widely used in areas such as game playing, robotics, navigation, and real-time decision making.

2. Deep learning (DL)—DL is also a subset of ML which is continuously and rapidly expanding to constitute its own field of AI. The artificial neural network algorithms used in DL are inspired by the structure and function of the human brain and are particularly useful in processing large and complex datasets (Fig. 9). DL models are composed of multiple processing layers for learning representations of data with multiple levels of abstraction (LeCun et al. 2015). A unique feature of DL is that performance of the model continues to increase with the addition of more data. DL is behind the advancements in image recognition, natural language processing, and speech recognition which can be used in applications like disease diagnosis from radiology images or developing speech recognition systems for patients with speech impairments.
3. Federated Learning—A federated machine learning approach allows models to be trained across numerous devices or servers holding local data samples, without exchanging the data itself. This approach ensures data privacy as all the raw data remain on the original device or server. The central server only receives updates to the model parameters, not the actual data, which is especially useful in scenarios where data privacy and security are critical.
4. Natural Language Processing (NLP)—NLP is a subfield of AI focused on the interaction between computers and humans in natural language. It can be used to mine valuable information from scientific literature, clinical records, and patient interactions, helping life scientists draw new insights or aid decision-making processes.
5. Network effects—In data science, network effects refer to the concept that the more data an algorithm has access to, more effectively it can learn patterns, make accurate predictions or uncover insights.
6. Bioinformatics—This is an interdisciplinary field that applies computational methods to analyze large collections of biological data, such as genetic sequences. AI can significantly speed up data processing and analysis in bioinformatics, providing insights into disease mechanisms, drug targets, toxicological effects, etc.
7. Drug Discovery—AI is revolutionizing the drug discovery process by predicting how different drugs will interact with targets in the body. Machine learning algorithms can process massive amounts of data on molecular structures and biological processes to help predict drug outcomes and side effects.
8. Genomics and Precision Medicine—AI is being used to analyze large genomic datasets to understand disease mechanisms at the molecular level, paving the way for personalized treatments. This has significant implications for diseases like cancer, where treatment can be tailored to the patient's specific genetic makeup.
9. Medical Imaging and Diagnosis—AI can analyze medical images like MRIs, X-rays, and CT scans, and detect anomalies, assisting doctors in diagnosing diseases like cancer, heart disease, and neurological disorders at an early stage. Digital pathology applications of AI may provide more reproducible and objective assessments of complex pathophysiology, morphological biomarker detection, patient stratification, and toxicological study outcomes (Baxi et al. 2021; Song et al. 2023).
10. Digital Twins – Large patient populations are being combined with systems biology models to create digital twins. Patient-specific digital twins can use information about an individual in combination with AI to parameterize models and predict individual susceptibilities or targeted therapeutic interventions.
11. Synthetic Data – Algorithms such as generative adversarial networks can be used to generate synthetic datasets that are trained from real world data and may supplement or substitute the need for experimental animals, e.g. via virtual control groups.
12. Large Language Models (LLMs) – LLMs are characterized by their extensive data training and high computational resource use. These models have billions of parameters and have evolved to achieve general-purpose language understanding and generation, with comprehensible textual reasoning and inference outputs. Models such as ChatGPT are trained on massive-scale data (i.e. the content of the internet), but increasingly LLMs are being trained for discipline-specific applications using quality-controlled datasets.

**Fig. 10:**
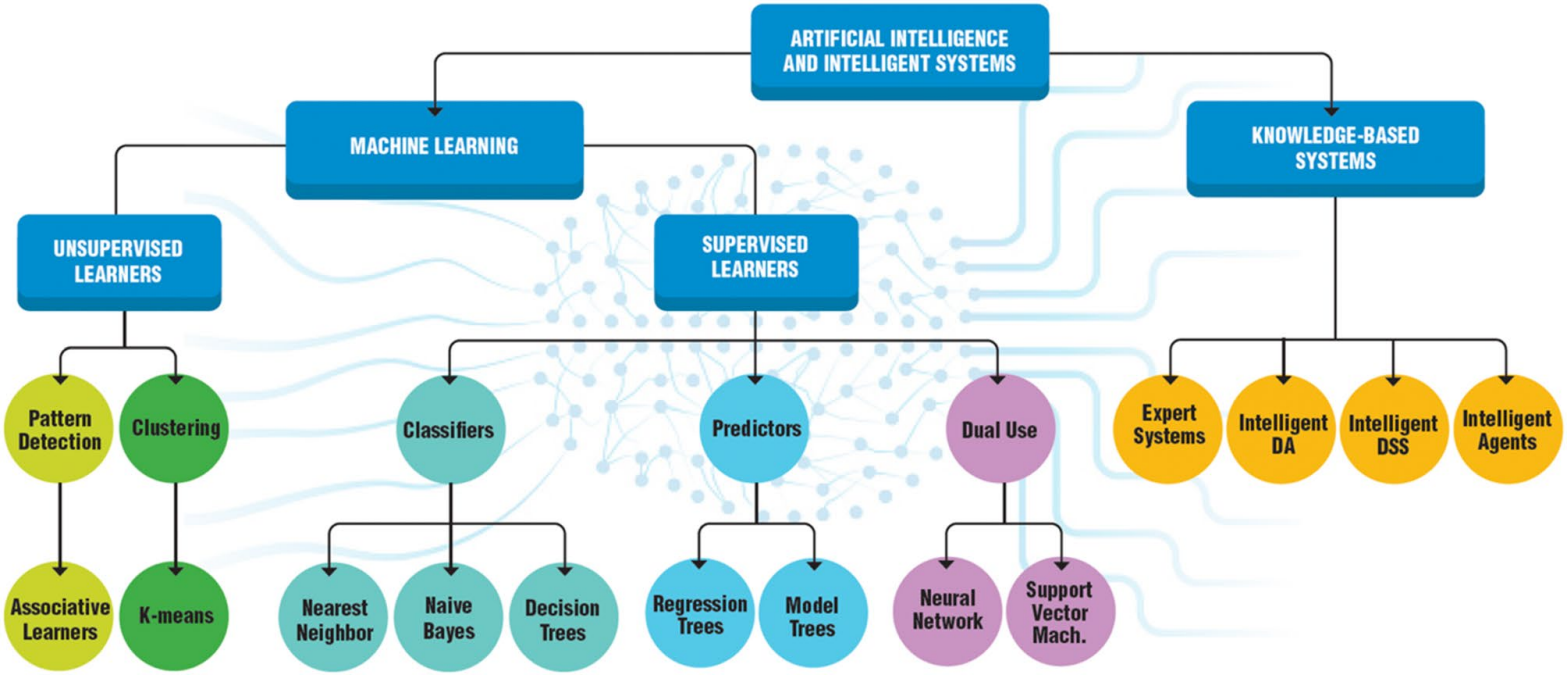
An Artificial Intelligence (AI) family tree. Modified and redrawn from https://ro.pinterest.com/pin/611645193133116162/

The multitude and continuously expanding universe of mathematical approaches used in AI is beyond the scope of this review. Figure 10 shows an “AI family tree”. The fundamentals of many of today’s algorithms have been around for decades, but only recently do we have enough data and sufficient computational power to make these technologies work.

## The evolution of AI in toxicology

Artificial intelligence (AI) and toxicology have evolved as distinct scientific disciplines over the past several decades. While toxicology has its roots in traditional observational studies, the data explosion, especially from high-throughput assays and omics technologies, has created new opportunities for applying AI techniques. The synergistic integration of these two fields is poised to transform how chemical toxicity evaluation is performed. This section provides an overview of the major developments at the intersection of AI and toxicology over the past few decades, in the order in which they first occurred. It highlights key milestones reflecting the evolution in capabilities, focus areas, and techniques as these fields have matured. The limitations faced during different eras as well as emerging trends for the future are discussed.

### Early expert systems (1980s–1990s)

The earliest applications of AI in toxicology involved expert systems, which aimed to encode human expert knowledge into computer programs using rule-based logic and reasoning. Programs like DEREK, METEOR, HazardExpert and OncoLogic were developed to predict potential toxicity based on chemical structure patterns and rules identified by experts (Marchant et al. 1988; Payne et al. 1995; Benfenati and Gini 1997). However, these systems were limited by the rigidity of rule-based approaches and the challenge of comprehensively encoding expert knowledge from literature, guideline studies, and human intellect. They relied heavily on manual input and lacked adaptability to new data. These limitations prevented wide adoption, but the efforts demonstrated promise for complementing human reasoning with prediction tools.

### Statistical learning and QSARs (1990s–2000s)

**Fig. 11:**
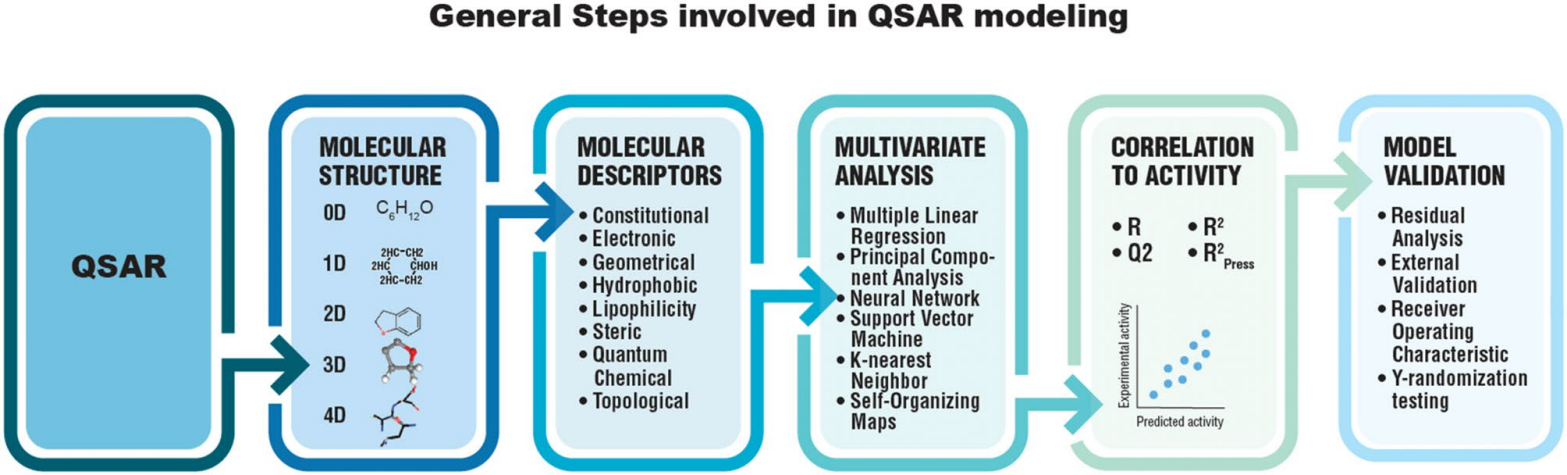
General steps involved in QSAR modelling. Modified and redrawn from (Abuhammad and Taha 2015)

The emphasis shifted from knowledge engineering to statistical and machine learning models driven by data. Quantitative structure–activity relationship (QSAR) models incorporated techniques like regression, random forests, and support vector machines to relate chemical descriptors to toxicity endpoints (Eriksson et al. 2003; Tropsha 2010) (Fig. 11). Public efforts like the OECD QSAR Toolbox (https://www.oecd.org/chemicalsafety/risk-assessment/oecd-qsar-toolbox.htm) compiled data and programs to generate QSARs for regulatory applications. However, reliance on human-crafted descriptors, simplistic models, and small datasets restricted predictive performance. This era evidenced growing recognition of the need for curated public data resources to develop robust data-driven toxicology tools. QSAR modeling continues to evolve with the advent and implementation of chemical curation workflows (Mansouri et al. 2024), more informative structural descriptors (Sedykh et al. 2020), large curated datasets (Karmaus et al. 2022; Kleinstreuer et al. 2016a), and ensemble modeling approaches (Mansouri et al. 2021).

### Data-driven movement (2000s–2010s)

The early 2000s marked the emergence of public repositories like PubChem, ChemBL, ACToR and Tox21/ToxCast that compiled volumes of chemical data and high-throughput screening assay results (Kavlock et al. 2012; Williams et al. 2017). Toxicogenomics data from microarrays reflected a shift from descriptive to mechanistic toxicology. These diverse evidence streams necessitated moving from statistical QSAR models to techniques like machine learning capable of integrating multi-modal data. An analysis of QSAR literature 2009–2015 (Devinyak and Lesyk 2016) observed that the number of QSAR papers using standard regression tools was decreasing, while more papers used ML methods, especially RF and naive Bayes. The Toxicology in the 21st Century initiative emphasized evidence-based, mechanism-driven predictive toxicology enabled by such data-rich resources (NRC 2007).

### Rise of deep learning (2010s-present)

The current era is being shaped by deep learning, which utilizes neural networks with multiple layers to extract higher-level features from raw input data. Seminal applications used deep learning on chemical structures to predict toxicity, outperforming previous approaches (Mayr et al. 2016; Luechtefeld et al. 2018a, b). DeepTox demonstrated the power of deep learning for diverse endpoints using Tox21 datasets (Wu et al. 2018). Active areas of research include multi-modal deep learning to integrate heterogeneous data types, generating synthetic toxicology data, and improving model interpretability. Initiatives like Tox21 and ToxCast continue generating rich public datasets to further AI capabilities (Kavlock et al. 2012).

### Emergence of AI for toxicology (present onwards)

Modern toxicology is increasingly embracing the synergies of big data and AI. Techniques like natural language processing are automating literature mining for evidence extraction. AI is also enabling integration of ontologies, adverse outcome pathways and systems models to derive mechanistic insights from multifaceted data. Cloud platforms are allowing easy access to computational toxicology resources. As predictive toxicology relies increasingly on in silico tools, AI has penetrated every stage of the toxicity testing paradigm. Explainable AI, automated lab robots, and sensors are expected to be transformative as data generation and analysis become more intertwined.

The trajectory of progress in applying AI for predicting and understanding chemical toxicity has closely mirrored advances in data availability, algorithmic capabilities, and interdisciplinary collaborations. While rule-based expert systems were promising proofs-of-concept, the lack of large curated datasets was a fundamental limitation. Deep learning and modern AI finally offer the potential to handle the complexities of toxicology big data and provide robust predictive tools to augment human insight. Federated learning across distributed servers is an opportunity which seems to be ideally suited for toxicology, where data sharing is often challenging. Promoting open data sharing and/or federated model building, encouraging academia-industry partnerships, and fostering AI literacy among toxicology researchers will be vital to fully realize the promise of this integrative new field – ToxAIcology (2023b, 2023c).

## AI for toxicity prediction

Predicting potential toxicity and adverse effects of chemicals is a crucial application of computational methods in toxicology. Traditionally, quantitative structure–activity relationship (QSAR) models have been used for this purpose. However, in some instances these models have limited predictive power as they rely solely on chemical descriptors and lack capacity to integrate diverse data types (Hartung and Hoffmann 2009). The field of computational toxicology has enjoyed rapid growth over the last decade, with the maturation of cognitive algorithmic tools and software to mine, process, and model data to facilitate robust and reliable predictions of chemical property, activity, and toxicity endpoints. Success lies in iterative and mutually informative approaches along a continuum of findable, accessible, interoperable, and re-usable (FAIR) data resources, predictive analyses, experimentation, and mechanistic models, with the goal of generating insights into human disease processes and their susceptibility to environmental perturbations. Underpinning this “CompTox continuum” is a multidisciplinary field that leverages big data and computational tools to join techniques of AI, ML, natural language processing, mathematical modeling, and data analytics to enhance and support human intellect. Applications to predictive toxicology range from model development for specific toxicity endpoints, to automation of data curation and annotation, to computational workflows enabling hypothesis generation and testing, to establishing scientific confidence in new approach methodologies. AI methods, especially DL, are a critical tool along the CompTox continuum to offer more robust solutions for predictive toxicology. With sufficient training data, DL models can capture complex relationships between chemical structure, bioactivity and toxicity. Various types of neural networks, like convolutional and recurrent neural networks, have been applied for toxicity prediction. One seminal study demonstrated the use of DL for predicting mutagenicity, reproducing the accuracy of mutagenicity assays for over 90% of chemicals (Mayr et al. 2016). Deep neural networks have also shown promise in predicting developmental and reproductive toxicity, such as teratogenicity (Challa et al. 2020; Sukur et al. 2023).

A key advantage of DL in toxicology is the capacity to learn both chemical structural features as well as patterns in assay bioactivity data predictive of toxicity. This ability to fuse heterogeneous data allows for more robust toxicity predictions. Deep learning methods are also better suited to handle large and complex toxicological datasets. Importantly, DL models can provide probabilistic predictions conveying the confidence of toxicity potential rather than binary or categorical classifications. However, reliance on large training datasets, lack of interpretability, and susceptibility to data biases remain as challenges. Ongoing research is focused on using explainable AI techniques to increase model interpretability (Jia et al. 2023). Overall, as chemically diverse and multi-modal toxicological datasets grow, AI and DL show immense potential to transform predictive toxicology.

## AI for toxicological data analysis

Modern toxicology research involves generating and analyzing large volumes of complex data from various sources. These include scientific literature, legacy animal studies, high-throughput screening assays, and diverse omics datasets. Manual curation and analysis of such heterogeneous big datasets is infeasible, as it is resource prohibitive and prone to human error. AI methods offer solutions through automating data extraction, normalization, annotation, integration and mining.

Natural language processing (NLP) techniques enable mining of unstructured textual data in published literature and old animal toxicity studies to extract relevant facts and relationships (Kleinstreuer et al. 2018; Foster et al. 2024). This can make use of existing evidence more efficient. For high-throughput screening data, AI can automate quality control, data cleaning, and hit-calling to streamline analysis (Allen et al. 2014).

Integrating diverse omics data to derive mechanistic insights is crucial but challenging for toxicologists. AI methods like graph neural networks can integrate transcriptomics, metabolomics and lipidomics data to enable multi-omics analyses (Wang et al. 2021). Through dimensionality reduction, AI can integrate such heterogeneous data modalities to find patterns predictive of toxicity phenotypes.

Causality assessment from observational data is another active area of AI research with applicability in toxicology for deriving adverse outcome pathways (AOPs) from diverse evidence (Rugard et al. 2020). Techniques like generative adversarial networks are also gaining traction to generate synthetic toxicology datasets where real data are lacking.

However, issues like hidden biases and batch effects in experimental data, reproducibility challenges, relevance of the training data for the prediction goals, and need for multidisciplinary expertise remain as caveats for applying AI in toxicological data analysis. Ongoing efforts like Tox21 are helping generate quality curated datasets to realize the promise of data-driven toxicology through AI.

## AI for risk assessment

Risk assessment is central to regulatory decision-making in toxicology. It involves integrating data across multiple levels of biological organization to determine the probability of adverse effects occurring under specific exposure conditions. AI is well suited for data-driven quantitative risk assessment due to its capacity for probabilistic modeling.

Most AI models provide predictive outputs as probabilities or confidence levels rather than binary classifications. This allows for capturing and propagating various uncertainties in the risk modeling workflow. AI can account for population variability by incorporating diverse exposure, toxicokinetic and toxicodynamic data (Zhang et al. 2019). This enables more refined probabilities and margins-of-exposure calculations. Bayesian approaches have become increasingly applied due to vast improvements in computational processing power and speed, providing probabilistic distributions that can be used to estimate risk and protect for sensitive and vulnerable subpopulations (Chiu et al. 2017).

AI can also help overcome challenges in extrapolating dose–response or exposure–response relationships from high-to-low doses typical in toxicology risk assessment. Deep learning models are capable of integrating diverse endpoint assays with in vivo data to derive more robust point-of-departure metrics for low-dose extrapolation (Thomas et al. 2019).

Active areas of AI research for risk assessment include integrating human and animal data, combining in vitro and in vivo data, and incorporating mechanistic or causal biological knowledge into probabilistic risk models. However, issues like model interpretability, uncertainty quantification, and bias management remain as challenges. Alignment with the adverse outcome pathway framework and OECD guidelines for quantitative in vitro-to-in vivo extrapolation is also warranted.

Overall, as toxicology progressively shifts from qualitative hazard identification to quantitative risk-based paradigms, AI adoption for predictive risk modeling will likely accelerate to strengthen evidence-based safety decision making.

## Explainable AI and toxicology

While AI models like deep neural networks achieve high predictive performance, their inner workings are often opaque making them hard to understand and interpret, and thus less likely to achieve regulatory acceptance and implementation. This black-box nature poses particular challenges for applications in toxicology where mechanistic transparency and causal explanations are crucial. The field of explainable AI (xAI) aims to address these interpretability issues (Samek et al. 2021).

xAI refers to methods for producing explainable models while also enabling human-understandable explanations to be generated for individual predictions. Strategies like visualizing activations in hidden layers, occlusion analysis, and perturbation-based approaches are being applied to unpack and demystify AI toxicity models.

Local explanation methods can determine the influence of different input chemical features on individual toxicity predictions. Global explanation techniques characterize the entire model behavior through surrogate models or summary visualizations. xAI implementations are being standardized through open-source libraries like InterpretML and initiatives such as DARPA's xAI program (Gilpin et al. 2018).

Most xAI approaches remain model-specific and focused on post-hoc explanations. Advances are needed for standardized, human-centered xAI techniques applicable across different model types and use cases in toxicology. Alignment with the adverse outcome pathway knowledge framework (OECD 2017) could be valuable for mechanistic and causal insights. Overall, xAI will be key for increasing trust and transparency in AI-based decision support systems for regulatory toxicology.

## Challenges and opportunities

While AI promises to be transformative for toxicology, there are certain challenges that need to be addressed. Andreas Bender in his comment^3^*“‘AI’ in Toxicology (*In Silico* Toxicology) – The Pieces Don’t Yet Fit Together*” coined the Anna Karenina Principle, adapted from Tolstoy:”*All happy molecules are alike; each unhappy molecule is unhappy in its own way.*” Some key limitations and mitigation strategies are discussed below:

1. **Data bias and quality** AI models are prone to reinforcing biases in training data which can lead to inaccurate predictions. Ensuring curated, representative, and unbiased datasets for model development is crucial (Tong et al. 2018).
2. **Lack of standardization** Heterogeneous data formats, protocols, and nomenclature make integration challenging. Community efforts for standardized ontologies, minimal reporting guidelines, and FAIR data practices are needed.
3. **Multidisciplinary expertise** Developing and implementing AI workflows require collaborating with data scientists, software engineers, chemists, biologists and toxicologists. Cross-domain partnerships should be fostered.
4. **Model interpretability** Complex AI models lack interpretability. Advances in explainable AI techniques to derive mechanistic understanding from models are needed.
5. **Regulatory acceptability** Lack of transparency around model assumptions and development could hinder regulatory endorsement. Verification, validation and uncertainty quantification are important. A recent report from the Interagency Coordinating Committee on the Validation of Alternative Methods (ICCVAM) offers a foundational framework that is general across new approach methodologies (ICCVAM 2023), while efforts at the OECD have generated a new QSAR Assessment Framework (OECD 2023).
  Despite these limitations, AI also presents promising opportunities such as:

1. **Animal replacement** I.e., reducing animal Testing: AI predictive models trained on existing data can reduce reliance on animal studies to screen new chemicals for toxicity.
2. **Accelerating safety evaluation** AI can automate tedious tasks allowing toxicologists to focus on high-value complex analyses to accelerate assessment.
3. **Democratization of knowledge** AI systems can enable easy access to toxicology prediction tools and databases to aid various end-users from regulators to industry. AI/big data and globalization are two sides of the same coin or probably better two sides of the same Rubik’s cube. Similar to science and enlightenment.
  The following section details specific subdisciplines and example projects that are effectively developing and applying AI to enhance predictive toxicology.

## AI to accelerate evidence-based toxicology

Evidence-based toxicology (EBT) (Hoffmann and Hartung 2006) aims to bring principles of transparency, objectivity, and consistency from evidence-based medicine to toxicology and risk assessment. Core EBT activities include systematic literature reviews, quality appraisal of studies, quantitative evidence synthesis, and evidence integration (Hoffmann et al. 2017). AI methods like machine learning and neural networks have potential to assist with and enhance various EBT tasks through automation. SysRev^4^ is such a tool allowing semi-automated systematic reviews (Bozada et al. 2021). AI capabilities for processing text, images, and diverse datasets can help extract, analyze and integrate evidence from toxicology literature and legacy reports. Neural networks can learn to assess study quality and bias. Meta-analysis and evidence synthesis algorithms operating on standardized data can accelerate quantitative synthesis. Knowledge graphs and interactive visualizations can provide evidence maps. Explainable AI techniques promote model transparency. Overall, purposeful AI design and use can both accelerate and enhance EBT.

**Fig. 12:**
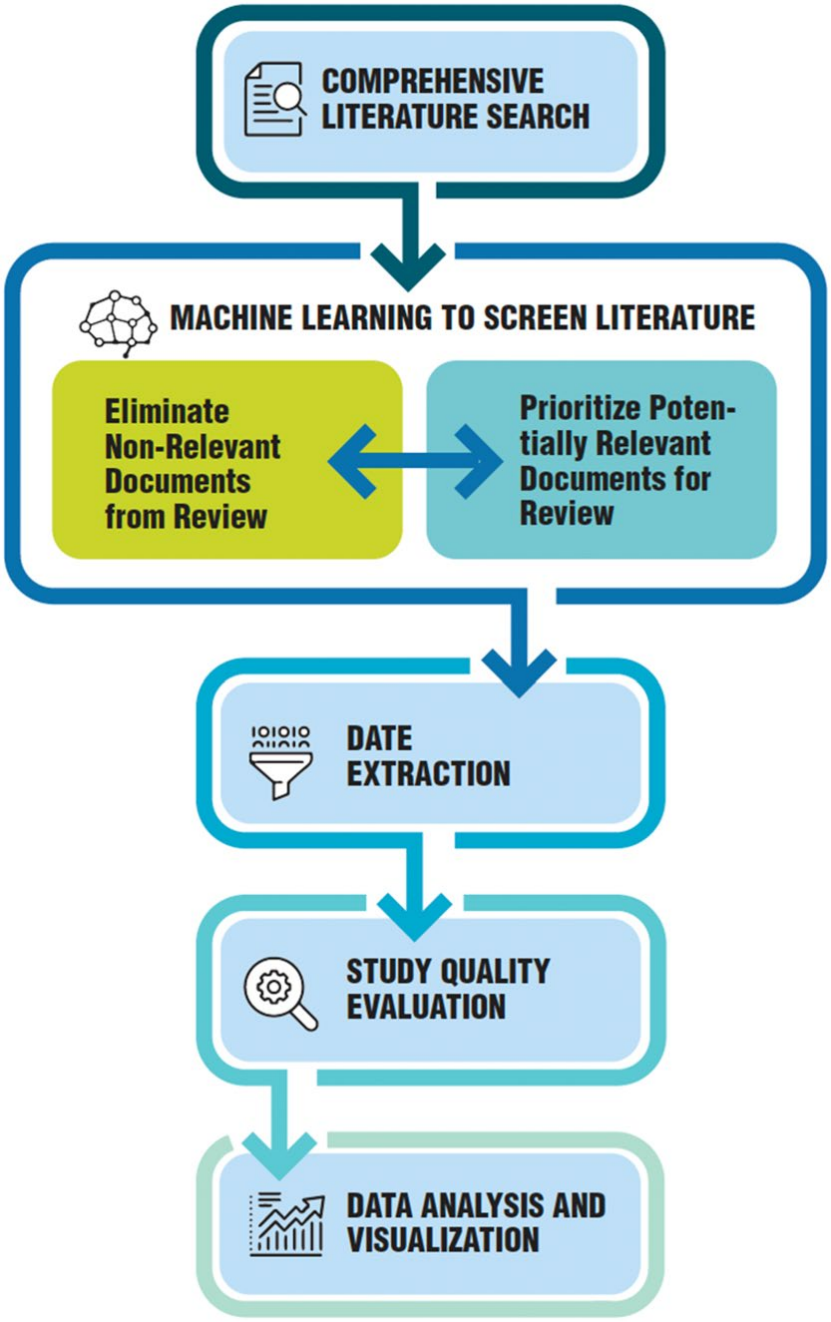
Machine learning in the systematic review process. Modified and redrawn from Varghese et al. (2020)

**Fig. 13:**
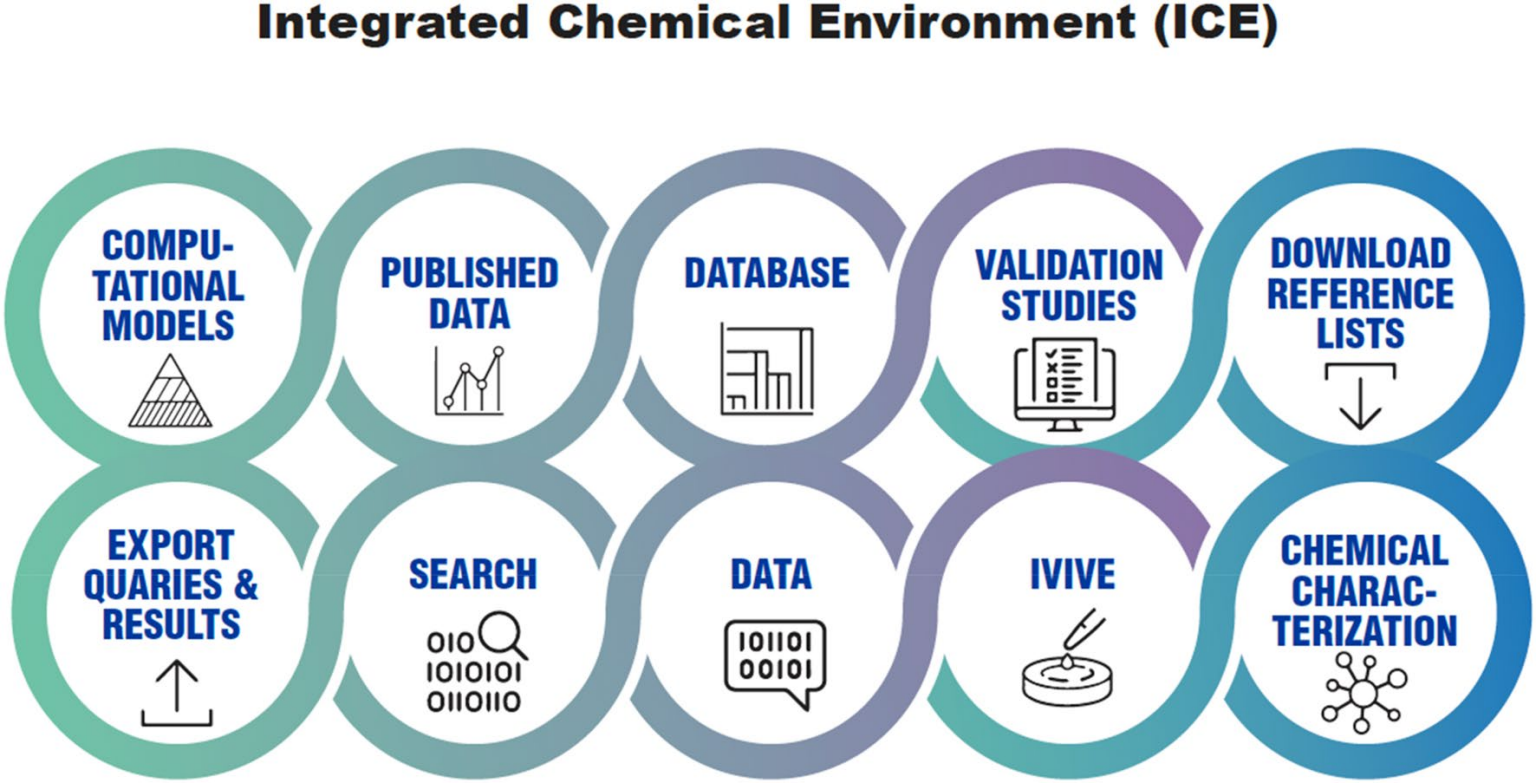
Main Components of the NIEHS Integrated Chemical Environment. IVIVE = In vitro-to-in vivo-extrapolation. Modified and redrawn from https://ice.ntp.niehs.nih.gov/

1. Automated Literature Mining—A fundamental challenge in EBT is comprehensive evidence gathering from enormous toxicology literature. AI text mining uses natural language processing (NLP) to extract facts, relationships, and reported findings from papers to populate structured databases (Yan et al. 2022). Toxicological ontology mapping further enables mining context-specific information (Foster et al. 2024). Legacy toxicity data in PDF reports can also be unlocked by AI optical character recognition and document classification techniques (Clark and Divvala 2016). Metadata extraction and reinforcement learning supports document triage and search (Walker et al. 2022). Thus, AI can accelerate evidence identification and extraction (Fig. 12).
2. Risk of Bias Assessment—Quality appraisal of studies for risk of bias is integral to EBT but time consuming. AI holds promise for automated study scrutiny using guidelines and checklists to train neural networks to automate the scrutiny of AI-based studies, ensuring that they meet the necessary standards for transparency, completeness, and quality. Models can learn risk-of-bias patterns from manual assessments done by experts. AI-human loops can enable efficient semi-automated study appraisal Fig. 13.
3. Evidence Synthesis—Quantitative evidence synthesis involves aggregating results across similar studies, requiring data normalization and statistical meta-analysis. AI can automate extraction of study results and metadata into standardized formats (Kiritchenko and Mohammad 2017). Meta-analysis algorithms tailored for toxicology data can synthesize diverse evidence types (Vidgen et al. 2021).
4. Uncertainty Analysis—Characterizing uncertainties is key in EBT evidence synthesis. Bayesian AI models can quantify uncertainty bounds for meta-analysis while sensitivity analysis based on neural network Jacobian matrices can reveal influential sources of uncertainty (Kwon et al. 2020; Liu et al. 2023; Li et al. 2021). This supports focus on material gaps.
5. Evidence Maps—AI knowledge graphs integrating literature mining and network analysis techniques can provide interactive visualizations of evidence clusters and relationships (Kejrival 2022). User filters and multi-dimensional views allow exploring evidence maps tailored to specific questions.
6. Explainable AI—Transparency of AI models and analyses is crucial for trustworthy EBT integration. Explainable AI methods like LIME (Ribeiro et al. 2016) can provide local explanations for individual predictions. Model debugging techniques including adversarial examples, counterfactuals and stress testing evaluate model limitations (Chou et al. 2022). Causal analysis ensures internal validity.

In summary, AI has considerable potential to accelerate and enhance EBT by extracting, analyzing, synthesizing and integrating toxicological evidence from literature and data. This can overcome manual bottlenecks in evidence-based approaches. However, purposeful and ethical AI design remains pivotal for reliable and transparent EBT assisted by AI. Overall, AI transformation coupled with ongoing efforts in mechanistic toxicology will enable more swift, robust and reproducible evidence-based safety evaluations.

## AI to enable probabilistic risk assessment

Probabilistic risk assessment (ProbRA) has emerged as a quantitative methodology to characterize risks by incorporating probability distributions instead of single point estimates for key model parameters. This captures inherent variability and uncertainties to enable more realistic risk estimates compared to traditional deterministic approaches (Maertens et al. 2022). AI methods are well suited for facilitating and enhancing various aspects of ProbRA, given their ability to analyze large, multi-modal datasets, identify patterns, and make data-driven predictions. This chapter provides an overview of some key application areas where AI shows promise to transform ProbRA capabilities by automating tedious tasks, integrating diverse evidence streams, explaining model behaviors, and supporting risk management decisions under uncertainty. However, responsible and ethical development and use of AI tools remain crucial for wider acceptance of data-driven ProbRA.

1. Data Extraction and Curation—The first step in evidence-based ProbRA involves comprehensive gathering and curation of relevant data from various sources to parameterize risk models. AI natural language processing (NLP) methods enable text mining of enormous scientific literature to extract factual information and reported evidence in toxicology (Hoffmann et al. 2017). For example, Grover et al. (2019) designed a Biomedical Entity Relation Extraction dataset spanning scientific findings from over 18,000 PubMed abstracts annotated via crowdsourcing. Such abilities to rapidly compile relevant prior results can better inform ProbRA models. Meanwhile, vast volumes of historical toxicity data exist as inaccessible legacy reports in PDF formats. AI optical character recognition (OCR) and Intelligent Document Processing (IDP) allow layout understanding techniques that unlock these by extracting tables, figures, and textual information (Acodis 2020). Further, meta-analysis algorithms and study quality assessment models can synthesize evidence from diverse publications to derive realistic model parameter estimates and uncertainties (Briggs et al. 2012). For big datasets, which are commonplace in chemical risk assessment, AI methods like deep learning enable automated data wrangling, normalization and quality checking to maintain reliability (Luo et al. 2021). Overall, AI unlocks large volumes of multimodal data to better parameterize ProbRA models.
2. Predictive Modeling—At the core of ProbRA are mathematical models relating exposures to eventual risks. AI methods like deep neural networks have shown tremendous successes for predictive toxicology, for example, deep learning on chemical structures and bioactivity data can yield high accuracy models for mutagenicity, rodent carcinogenicity, and other toxicity endpoints suitable for ProbRA (Mayr et al. 2016). Multimodal deep learning suits ProbRA needs by jointly analyzing chemical information, in vitro assay data, omics profiles etc. to derive robust structure–activity relationships capturing complex real-world risks (Wu et al. 2018). Deep generative models like generative adversarial networks can create realistic synthetic biomonitoring data where human evidence is sparse, to better train ProbRA models (Kadurin et al. 2016). Overall, AI predictive modeling strengthens the evidence-basis of ProbRA.
3. Uncertainty Quantification—A key advantage of ProbRA lies in expressing model predictions along with associated uncertainties that can guide further refinement. AI methods like Bayesian deep learning explicitly model uncertainties in neural network weights and outputs (Kwon et al. 2020). This allows deriving confidence intervals and probabilities for risk estimates. AI uncertainty quantification techniques also enable efficient sensitivity analysis to identify influential model parameters (Liu et al. 2020). This supports focusing additional data curation on the most important sources of uncertainty.
4. Evidence Integration—Integrating diverse evidence streams from literature meta-analysis, physicochemical data, in vitro & in vivo assays, etc. to derive weight-of-evidence risk conclusions is challenging. AI provides suitable tools such as probabilistic logic learning methods to synthesize disparate pieces of evidence (Vidgen et al. 2021). Causal analysis with Bayesian networks can incorporate mechanistic understanding into probabilistic inferences (Korb and Nicholson 2008). Overall, AI enables aggregating population variability, human heterogeneity, exposure factors and hazard potential into integrated ProbRA models.
5. Explainable AI—While AI models provide powerful predictive capabilities, their ‘black box’ opacity poses challenges for regulatory acceptance in particular for risk assessment applications. The xAI field aims to decipher model behaviors and predictions (Adadi et al. 2018). For instance, methods like integrated gradients can identify influential chemical features driving toxicity predictions (Vidgen et al. 2021). Such transparency will be key where ProbRA informs high-stakes decision making. Evaluation of model limitations via testing on out-of-distribution data will also be crucial.

In summary, AI has immense potential to enable more expansive, transparent and human-centric ProbRA by automating data extraction & curation, predicting risks from multimodal data, quantifying uncertainties, integrating diverse evidence, and explaining model behaviors. This can lead to higher quality risk-based decisions to better safeguard human and environmental wellbeing. However, responsible AI development and use by multidisciplinary teams spanning AI, risk science and domain experts will be pivotal for wider acceptance and realizing the promises of AI-enabled ProbRA.

## AI to enable the ONTOX project goals

AI techniques offer immense potential to facilitate various aspects of the goals of numerous global collaborations, e.g. ONTOX, RISK-HUNT3R, PANORAMIX, PrecisionTox, and aligns with the paradigm shift towards human-relevant new approach methodologies (NAMs) and evidence-based toxicology. Efforts under these projects enabled by AI include extracting and analyzing evidence from literature, generating predictive models from chemical and biological data, characterizing uncertainties, integrating diverse evidence streams in a causal framework, and promoting model transparency. The ONTOX project funded by the EU Horizon 2020 program aims to develop innovative non-animal methods (NAMs) for predicting repeat dose systemic toxicity. The specific adverse outcomes addressed are liver steatosis and cholestasis, kidney tubular necrosis and crystallopathy, and neural tube closure and cognitive function defects. A key objective is integration of tailored exposure assessment with mechanistic information to enable human-relevant chemical safety evaluation (Vinken et al. 2021).

1. Data Curation and Extraction—A comprehensive evidence-base of existing knowledge is key to develop NAMs for predicting toxicity pathways and outcomes. AI text mining of enormous scientific literature can accelerate evidence gathering. For instance, natural language processing methods developed by Corradi et al. (2022) could extract factual toxicological findings and relationships from PubMed abstracts and full texts. Further, AI techniques can unlock legacy toxicity data trapped as scan PDF reports via optical character recognition and document layout analysis (Palm et al. 2019). Also, standardized ontologies mapped to adverse outcome pathways will enable structured representation of extracted evidence (Kleinstreuer et al. 2016a, b, c). Overall, AI supports ONTOX goals by better utilizing existing knowledge.
2. Predictive Modeling—Modern deep learning models excel at learning predictive patterns from chemical and biological data relevant for toxicity prediction. For example, graph neural networks operating on molecular graphs can yield high accuracy for toxicity endpoints (Jin et al. 2019). Integrating such chemical data with pathway-based biological readouts using multimodal deep learning suits ONTOX’s goals of mechanistic NAMs. Where human evidence is scarce, deep generative models can create realistic synthetic data (Kadurin et al. 2016) to train more robust AI models and analyze uncertainties. Causal analysis methods can infer plausible mechanisms from observational data (Schölkopf et al. 2021). Overall, AI modeling strengthens ONTOX’s evidence basis.
3. Uncertainty Characterization—For wider regulatory acceptance, NAMs must provide confidence estimates alongside predictions. AI tools like Bayesian deep learning (Kwon et al. 2020) allow probabilistic outputs to capture uncertainties. Sensitivity analysis based on automatic differentiation methods (Daxberger et al. 2021) can reveal the most influential sources of uncertainty to prioritize additional experiments or data curation. Thus, AI facilitates uncertainty characterization in ONTOX.
4. Evidence Integration—Integrating diverse evidence streams from literature mining, in vitro assays, omics data, etc. into a consistent picture is challenging. AI techniques like probabilistic logic learning using Markov Logic Networks (Richardson and Domingos 2006) allow evidence synthesis by incorporating domain knowledge. Causal graph representations permit incorporating mechanistic insights into model predictions. Overall, AI enables evidence integration for ONTOX.
5. Explainable AI—While AI models provide strong predictive performance, transparency regarding their reasoning and potential limitations is crucial for trust and regulatory acceptance. Explainable AI techniques, such as layerwise relevance propagation for deep learning models (Bach et al. 2015), can identify influential features driving particular predictions. Evaluation on out-of-domain examples can reveal model vulnerabilities. Such AI model debugging ability aids ONTOX’s goals.

In summary, AI has an essential role in extracting, analyzing and integrating evidence to develop robust NAMs for human-relevant chemical safety assessment as aimed by ONTOX and other global projects. However, purposeful and ethical AI development and use remains pivotal for realizing its benefits. Overall, AI transformation coupled with ongoing efforts in mechanistic toxicology will enable the ONTOX vision of evidence-based chemical safety assessment using fewer animals.

## The example of the Integrated Chemical Environment (ICE) as resource and user interface for AI in toxicology

The Integrated Chemical Environment (ICE: https://ice.ntp.niehs.nih.gov/) is an example of a suite of models, tools, and high-quality datasets that are designed to democratize access to AI-enabled computational toxicology resources across the scientific community (Abedini et al. 2021; Daniel et al. 2022). The ICE database is organized by toxicity endpoint and mechanism, and uses standardized terminology, units, and formatting to adhere to FAIR principles, and is programmatically accessible via a REST API. Additional curated information in ICE includes reference chemical lists with classifications and bioactivity, in vitro assays annotated with defined terminology and curated based on analytical QC and technological information, and computational workflows. ICE facilitates exploration and interpretation of large datasets like the Tox21 HTS human cell-based assay data, by linking assay targets to organ systems in the body and mechanisms of toxicity. The user can interact with the concentration response data from individual tests or combine data from mechanistically related assay targets to provide insight into biological effects observed at increasing exposures to environmental toxicants. Data-driven graphical displays provide the ability to filter the in vitro HTS data based on a variety of parameters like mechanistic target, platform, and bioactivity level, and search for similar chemical structures using chemical identifiers, SMARTS strings, Tanimoto scores, and availability of bioactivity data.

Other AI-enabled computational tools in ICE can be used to run virtual animal/human studies, via user-defined parameters like species, dosing route, duration, etc., to simulate how external chemical exposure will result in internal chemical concentrations in different tissues of the body (Hines et al. 2022). These PBPK models can be run in a forward dosimetry fashion, or in reverse dosimetry to perform in vitro to in vivo extrapolation (IVIVE), where critical parameters like plasma protein binding or hepatic clearance are either experimentally derived or predicted using embedded ML models. Additional workflows in ICE provide insight into where chemicals of concern appear in consumer products, cross-referenced with the bioactivity patterns across groups of mechanistic targets from the HTS data, to further link exposure to potential toxicity.

## The future of AI in toxicology

**Fig. 14:**
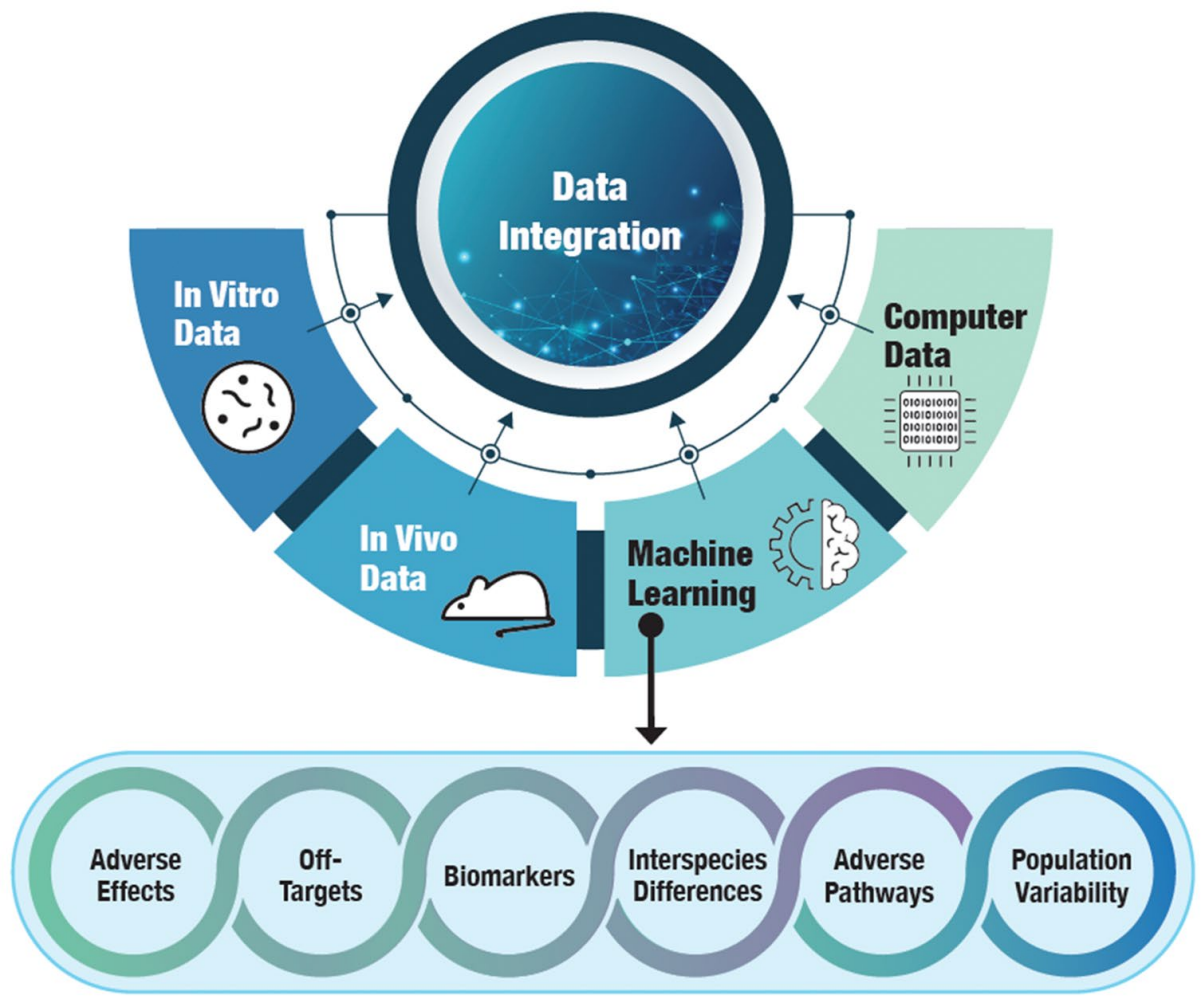
Evidence integration serving the different goals of safety sciences. Modified and redrawn from Vo et al. (2019)

The integration of AI in toxicology is still in its early stages. Overall, a responsible adoption of AI in toxicology research and regulation requires proactively addressing concerns around ethics, transparency, data practices and model interpretability while harnessing the power of AI. As techniques and adoption mature, AI is expected to become integral to multiple facets of toxicology. Figure 14 summarizes some of the use areas.

**Fig. 15:**
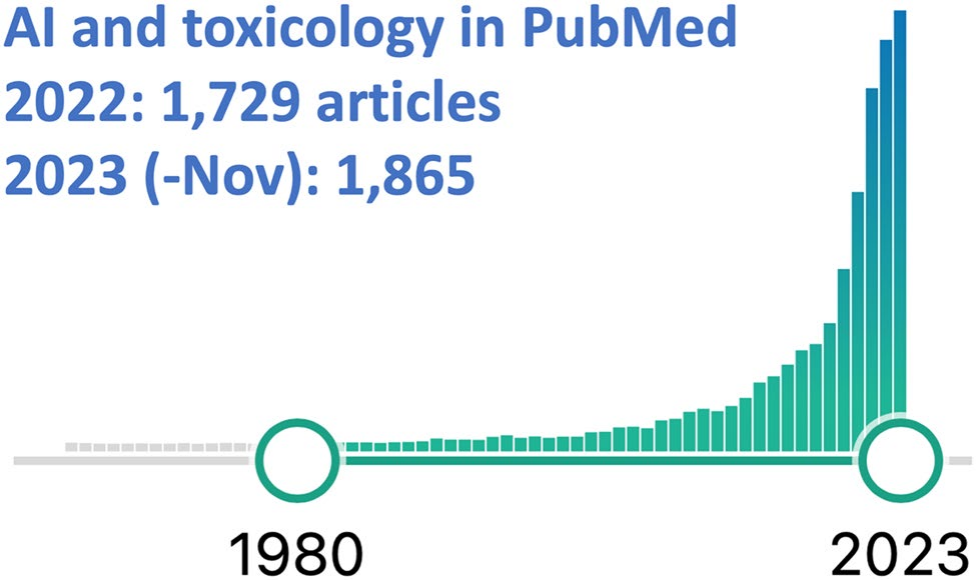
Articles in PubMed by year combining toxicology with AI. The following search was conducted in PubMed (https://pubmed.ncbi.nlm.nih.gov) (A.I. OR artificial intelligence OR machine learning) AND (toxicology OR toxicity OR hazard) on 28 Nov 2023

Toxicology has started to embrace AI as shown in Fig. 15 with the logarithmic growth of such articles combining the two from 1980 to now. Some key trends for the future of AI in toxicology are:

### Predictive toxicology

Increasing availability of diverse toxicological big data will drive more powerful and generalized predictive models using techniques like deep learning and reinforcement learning.

### Mechanistic toxicology

Causal inference methods and graph neural networks will derive explanatory networks integrating multiple evidence streams to refine mechanistic understanding and AOPs.

### In vitro to In vivo extrapolation

Multi-modal in vitro assay data will be integrated with PBPK modeling to enable robust quantitative IVIVE tailored to human biology for more accurate risk assessment.

### Toxicant screening

Automated high-throughput toxicity testing systems coupled with robotics and sensors will enable rapid screens for environmentally relevant mixtures guided by AI experimental design.

### Precision toxicology

AI analysis of exposure, genetic, epigenetic and microbiome data will enable personalized toxicity risk assessment and precision safety testing.

To realize this future, multidisciplinary and multi-sector collaborations are imperative. Ethical guidelines for transparency, bias mitigation, and responsible AI must be proactively developed through community engagement. Education programs integrating data science into toxicology curricula will develop the hybrid skillsets needed.

**Fig. 16:**
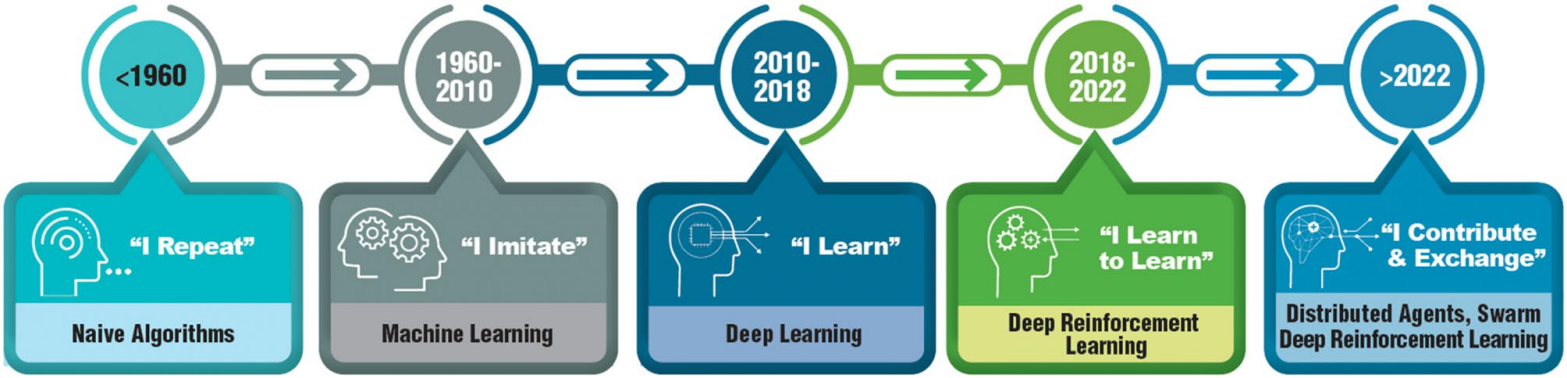
The evolution of AI approaches. Modified and redrawn from https://definingai.com/history-of-artificial-intelligence/

A recent workshop on the future of toxicology^5^ prompted by the US Department of Defense assembled an avant-garde of toxicologists and not surprisingly, AI played a central role in their vision (Hartung 2023a). With the rapid evolution of AI technologies (Fig. 16), current applications are already moving further to Distributed Agents and Swarm Deep Reinforcement Learning; part of this is federated model construction. While AI may automate certain tasks and enhance predictive capabilities, the creativity, skepticism and expertise of toxicology researchers will remain indispensable to employ these emerging tools for advancing the science of safety. AI holds great potential for life sciences, but it also brings challenges like data privacy and ethical concerns that need to be addressed responsibly. Moreover, the successful application of AI in life sciences requires interdisciplinary collaboration, bridging the gap between AI experts and life scientists.

## Data Availability

No datasets were generated in this study.

## Footnotes

1 https://www.weforum.org/press/2023/06/new-world-economic-forum-research-identifies-top-ai-applications-that-could-revolutionize-global-healthcare/.

2 https://www.weforum.org/reports/scaling-smart-solutions-with-ai-in-health-unlocking-impact-on-high-potential-use-cases.

3 http://www.drugdiscovery.net/2019/08/31/ai-in-toxicology-in-silico-toxicology-the-pieces-dont-yet-fit-together/

4 https://sysrev.com.

5 https://basicresearch.defense.gov/Portals/61/Documents/future-directions/Future%20Directions%20Workshop%20-%20Advancing%20the%20Next%20Scientific%20Revolution%20in%20Toxicology.pdf?ver=q0_CyJCAT-aj4HVv_W0a9Q%3D%3D.
